# Partial femoral head replacement: a new innovative hip-preserving approach for treating osteonecrosis of the femoral head and its finite element analysis

**DOI:** 10.3389/fbioe.2024.1352882

**Published:** 2024-01-23

**Authors:** Yanjiang Yang, Xiaodong Cheng, Wei Chen, Guimiao Li, Yuchuan Wang, Weiyi Sun, Wen An, Qi Zhang, Yingze Zhang

**Affiliations:** ^1^ Trauma Emergency Center, The Third Hospital of Hebei Medical University, Shijiazhuang, Hebei, China; ^2^ Orthopaedic Research Institute of Hebei Province, Shijiazhuang, Hebei, China; ^3^ Key Laboratory of Biomechanics of Hebei Province, Shijiazhuang, Hebei, China; ^4^ NHC Key Laboratory of Intelligent Orthopaedic Equipment, Shijiazhuang, Hebei, China; ^5^ Hebei Orthopaedic Clinical Research Center, Shijiazhuang, Hebei, China

**Keywords:** femoral head replacement, hip-preserving, osteonecrosis of the femoral head, finite element analysis, von Mises stress

## Abstract

**Purpose:** Controversy remains regarding the optimal treatment for stage III Osteonecrosis of the femoral head (ONFH). This study presents, for the first time, the precise treatment of stage III ONFH using the “substitute the beam for a pillar” technique and performs a comparative finite element analysis with other hip-preserving procedures.

**Methods:** A formalin-preserved femur of male cadavers was selected to obtain the CT scan data of femur. The proximal femur model was reconstructed and assembled using Mimics 20.0, Geomagic, and UG-NX 12.0 software with four different implant types: simple core decompression, fibula implantation, porous tantalum rod implantation, and partial replacement prosthesis. The finite element simulations were conducted to simulate the normal walking gait, and the stress distribution and displacement data of the femur and the implant model were obtained.

**Results:** The peak von Mises stress of the femoral head and proximal femur in the partial replacement of the femoral head (PRFH) group were 22.8 MPa and 37.4 MPa, respectively, which were 3.1%–38.6% and 12.8%–37.4% lower than those of the other three surgical methods.

**Conclusion:** The PRFH group exhibits better mechanical performance, reducing stress and displacement in the ONFH area, thus maintaining femoral head stability. Among the four hip-preserving approaches, from a biomechanical perspective, PRFH offers a new option for treating ONFH.

## Introduction

Osteonecrosis of the femoral head (ONFH) is a progressive multifactorial condition characterized by impaired blood supply and disruption of bone tissue synthesis within the femoral head (Zalavras and Lieberman, 2014a). The annual incidence rate of ONFH ranges from 7 to 20 per 100,000 individuals (Seijas et al., 2019). Causative factors include high-dose steroid use, alcohol abuse, and trauma. ONFH predominantly affects the weight-bearing zone of the femoral head, and if left untreated, it can lead to femoral head collapse and secondary hip joint osteoarthritis (Wu et al., 2020).

Multiple clinical guidelines have achieved consensus on the classification of ONFH based on the Association Research Circulation Osseous (ARCO) staging system, specifically recognizing stages I, II, and IV (Zhao et al., 2020; Association Related To Circulation Osseous Chinese Microcirculation Society, 2022). For stages I and II, treatment options such as core decompression, bone grafting, or osteotomy are recommended. Stage IV requires total hip replacement surgery. However, controversy remains regarding the optimal treatment for stage III ONFH.

Previous literature has documented the use of cartilage transplantation methods in the management of ONFH. However, these techniques have limited applicability in the femoral head due to its greater curvature, deeper location, and compromised blood supply compared to other joints such as the knee (Du et al., 2019). Some researchers propose that in addition to core decompression, the implantation of porous tantalum rods (Floerkemeier et al., 2011), non-vascularized fibular grafts (Feng et al., 2019), or vascularized fibular grafts (Zeng et al., 2013) may help prevent further cartilage collapse. However, neither approach provides robust structural support, satisfactory osseointegration, or a straightforward procedure (Korompilias et al., 2011).

To address these limitations, our research team has developed a partial femoral head replacement device for precise minimally invasive treatment of stage III ONFH according to the ARCO classification. This study aims to evaluate the biomechanical performance of the partial femoral head replacement device and establish a three-dimensional finite element model of ONFH. A comparative analysis was conducted with partial replacement of the femoral head (PRFH), core decompression (CD), core decompression with fibular grafting (CDFG), and core decompression with tantalum rod implantation (CDTRI) models.

## Materials and methods

This study received approval from the institutional review board (IRB) (NO. KE 2022-131-1) and complied with the Declaration of Helsinki.

### Three-dimensional modeling

A formalin-preserved femur of male cadavers was selected and underwent computed tomography scanning using a SOMATOM Definition AS scanner (Siemens, Germany). The scanning involved a slicing distance of 0.625 mm. A geometric model of the femur was then constructed using Mimics 20.0 software (Materialise, Leuven, Belgium). The non-uniform rational basis spline (NURBS) was created using Geomagic Studio 13.0 software (Geomagic Company, United States). The proximal femur’s solid model was meshed with C3D4 elements using Hypermesh 2014 software (Altair Company, United States).

### Establishing implants and assembled model

**FIGURE 1:**
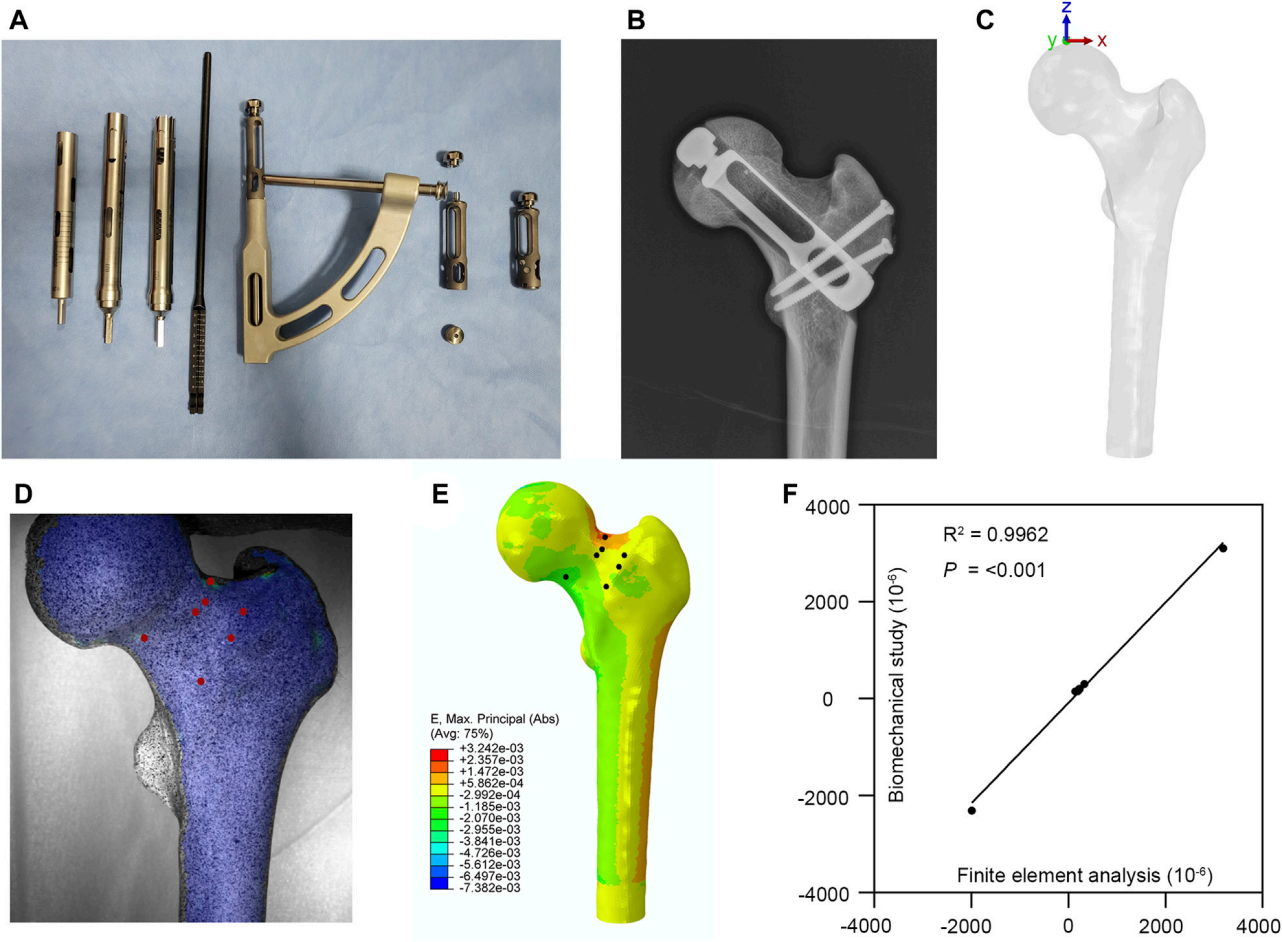
Partial replacement prosthesis and finite element model validation. **(A,B)** A partial replacement prosthesis. **(C)** The loading applied to the finite element model. **(D,E)** Comparison between the biomechanical test and finite element analysis. **(F)** Correlation analysis of finite element model validation.

**FIGURE 2:**
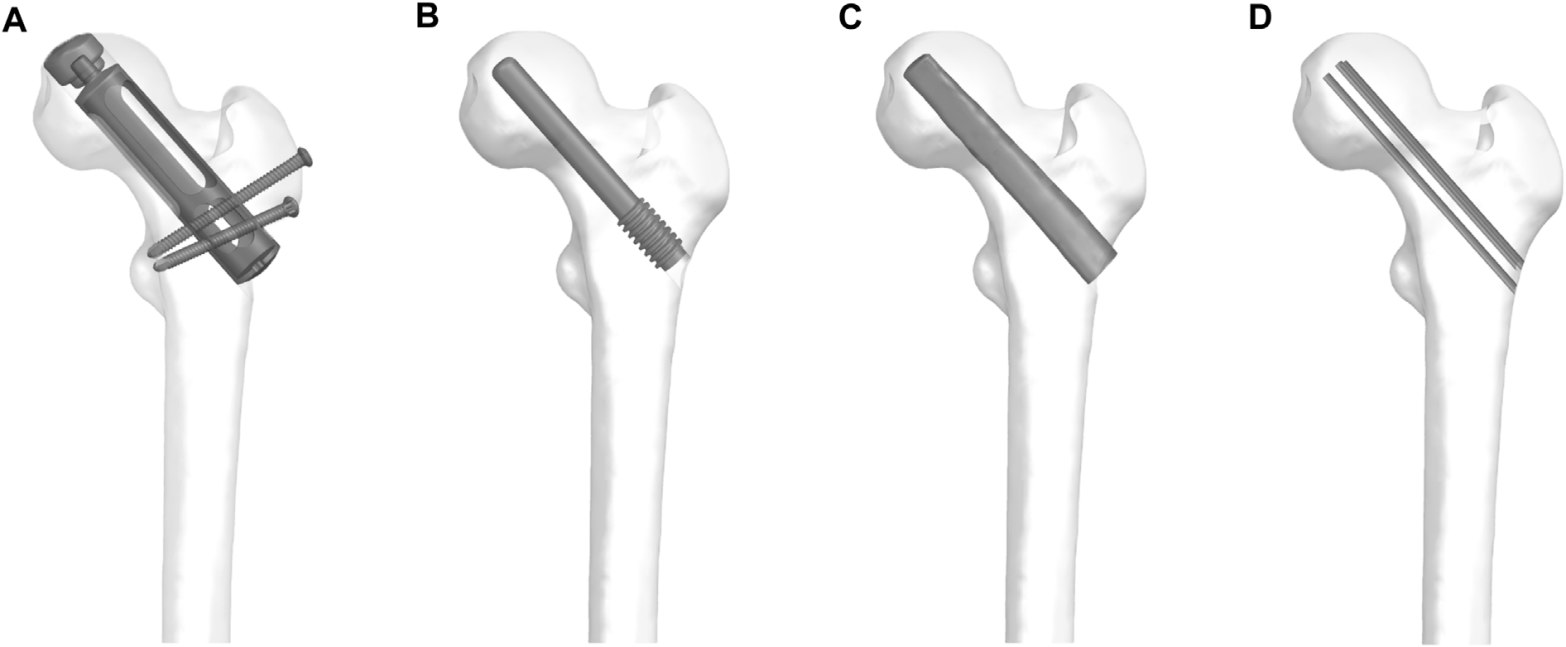
The proximal femur model with four types of implants: **(A)** partial replacement prosthesis, **(B)** porous tantalum rod implantation, **(C)** fibula implantation and **(D)** simple core decompression.

According to the prosthesis provided by Double Medical Technology Inc. (China) (Figures 1A, B), a model of a partial replacement prosthesis was created using UG-NX 12.0 software (Siemens Product Lifecycle Management Software Inc., United States). Utilizing UG-NX 12.0 software, we assembled the proximal femur model with four types of implants: simple core decompression, fibula implantation, porous tantalum rod implantation, and partial replacement prosthesis (Figure 2).

### Material properties and boundary conditions

The models were imported into abaqus 6.14 (Dassault Systèmes Solid Works Corp., Concord, MA, United States). Bone density is related to the material properties. Hence, the material properties of each femoral model were based on the Hounsfield units from the CT scan data (Reina-Romo et al., 2018). The mathematical formulas are as follows, where ρ was the bone density, HU represented the Hounsfield units, E was the modulus of elasticity, and *ν* was Poisson’s ratio: 1) ρ(g/cm^3^) = 0.000968*HU+0.5, 2) If ρ≤ 1.2 g/cm^3^; E = 2014ρ^2.5^ (MPa), *ν* = 0.2, 3) If ρ> 1.2 g/cm^3^; E = 1763ρ^3.2^ (MPa), *ν* = 0.32.

The partial replacement prosthesis was assigned as titanium alloy, with Young’s modulus (E) of 110,000 MPa and Poisson’s ratio (v) of 0.3. The fibula, tantalum rod, and cancellous bone were assigned to have different material properties, which were the same as those reported in other studies (Vail and Urbaniak, 1996; Louie et al., 1999; Liu et al., 2021). In these models, each part was assumed to be linear elastic, homogeneous, and isotropic. The moduli of elasticity were 15,100 MPa, 186,000 MPa, and 445 MPa, and the Poisson’s ratios were 0.3, 0.3, and 0.22, respectively (Huang et al., 2019; Xu et al., 2020). In this study, all models simulated the state of bone healing. There were no gaps around the interfaces between the grafted bone and femur in any of the postoperative models. It is important to note that in all the models, the necrotic site in the femoral head was uniformly considered as the weight-bearing region. Additionally, the necrotic tissue was removed and replaced with cancellous bone attributes.

Based on Bergmann’s research (Bergmann et al., 2001) on hip joint contact forces and gait, the forces acting on the femoral head were decomposed into three distinct forces of varying magnitudes and directions. Various movements were simulated to evaluate their influence on the stress encountered by the femoral head. Our finite element model is based on normal walking gait ({x, y, z} = {372, 224, −1,609} N) (Figure 1C). The forces applied to the femoral head were determined by the peak forces and directions observed during these activities.

The von Mises stress on the intact proximal femur was tested to analyze the mesh convergence. The convergence criterion used was a change of <5%. The mesh size was set to 1 mm. To emphasize the mechanical performance of the implant in the specific region of interest, a mesh size of 0.5 mm was employed for all implant components. The intact proximal femur was composed of 33,773 nodes and 158,985 elements. Table 1 showed the numbers of elements and nodes of four different models.

**TABLE 1:** Amounts of nodes and elements of four components.

Components	Nodes	Elements
PRFH	232,045	1,053,487
CDTRI	134,453	637,203
CDFG	61,167	282,853
CD	88,688	431,315

### Verification of finite element models

The femoral specimen after CT scanning was enveloped in polyethylene film and stored at −20°C until further use. Prior to the experiment, it was allowed to thaw at room temperature for 12 h. Subsequently, the soft tissues including muscles, periosteum, and ligaments were carefully excised. The midshaft of the femur, situated 25 cm from the femoral head, was surgically removed, and the proximal surface of the femur underwent meticulous polishing using fine sandpaper. To facilitate subsequent analysis, the specimens were uniformly coated with black and white matte paint. After the paint had thoroughly dried, the distal femur was firmly affixed within the module using denture powder. The ElectroForce 3330 Series II (TA Instruments, United States) was employed to apply axial pressure ranging from 0 to 600 N onto the surface of the femoral head at a rate of 5 N/s. Concurrently, the high-speed camera integrated within the GOM non-contact optical strain measurement system (GOM GmbH, Germany) captured the loading process at a frame rate of 7 frames/s. The resultant images were subsequently subjected to computer processing to derive strain images and quantify strain values specific to the proximal femur under an axial pressure of 600 N. Subsequent to data acquisition, the GOM Software 2021 was employed to select the appropriate starting point for calculations based on the collected images and to define the calculation area. Upon completion of the calculations, the strain cloud diagram was automatically generated. In the software interface, the stability-loaded cloud diagram was selected, and the strain values corresponding to the chosen points on the proximal femur were quantified (Figure 1D).

Under the same loading and boundary conditions as the biomechanical experiment, the maximum principal strain values at the corresponding position were calculated for the normal proximal femur finite element model (Figure 1E). The results indicate that our model is appropriate for the subsequent study (Figure 1F).

## Results

### Stress distribution

**FIGURE 3:**
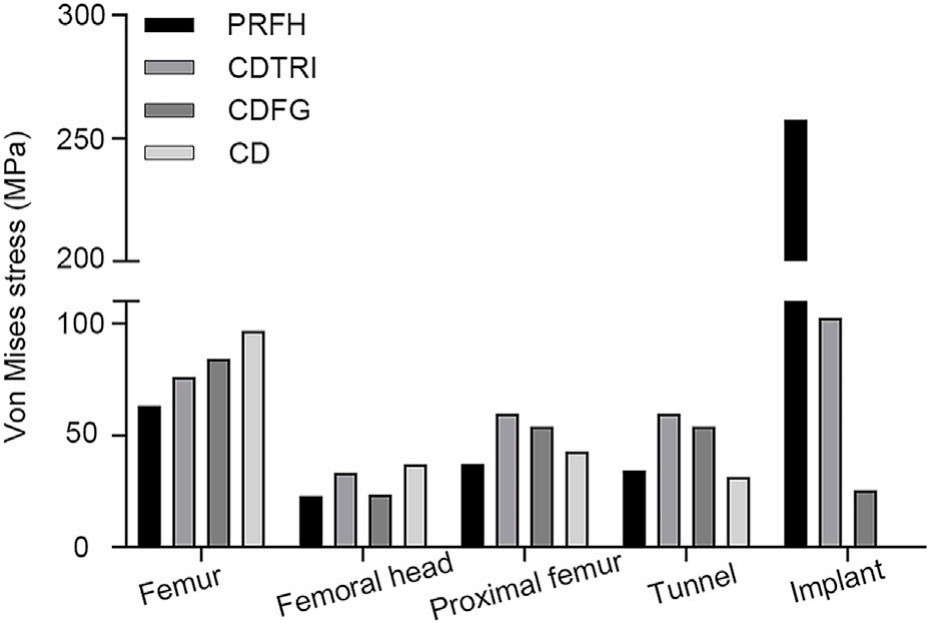
The von Mises stress distribution of different parts of femur in four models.

**FIGURE 4:**
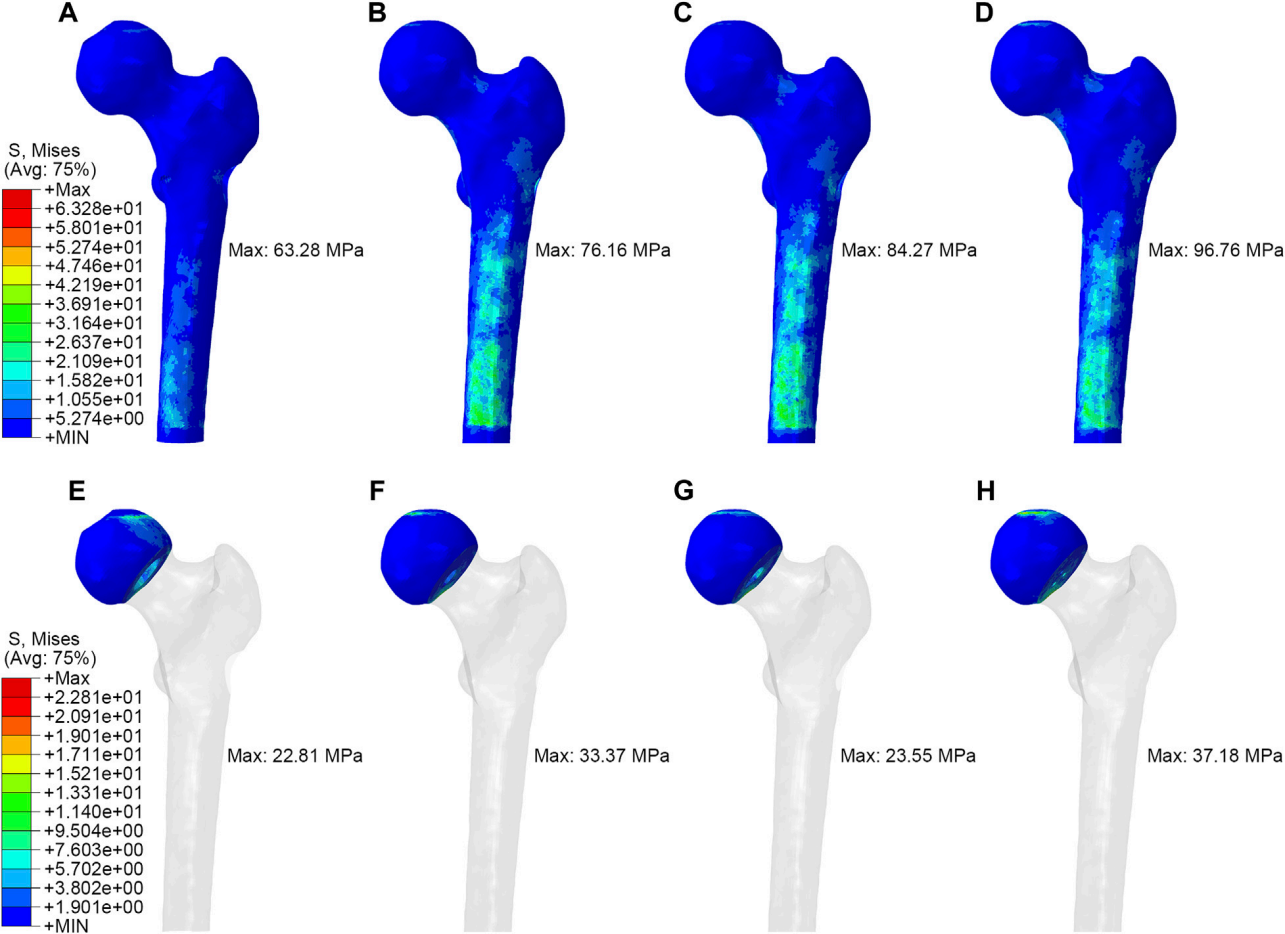
The von Mises stress distribution of whole femur and femoral head: **(A,E)** partial replacement prosthesis, **(B,F)** porous tantalum rod implantation, **(C,G)** fibula implantation and **(D,H)** simple core decompression.

**FIGURE 5:**
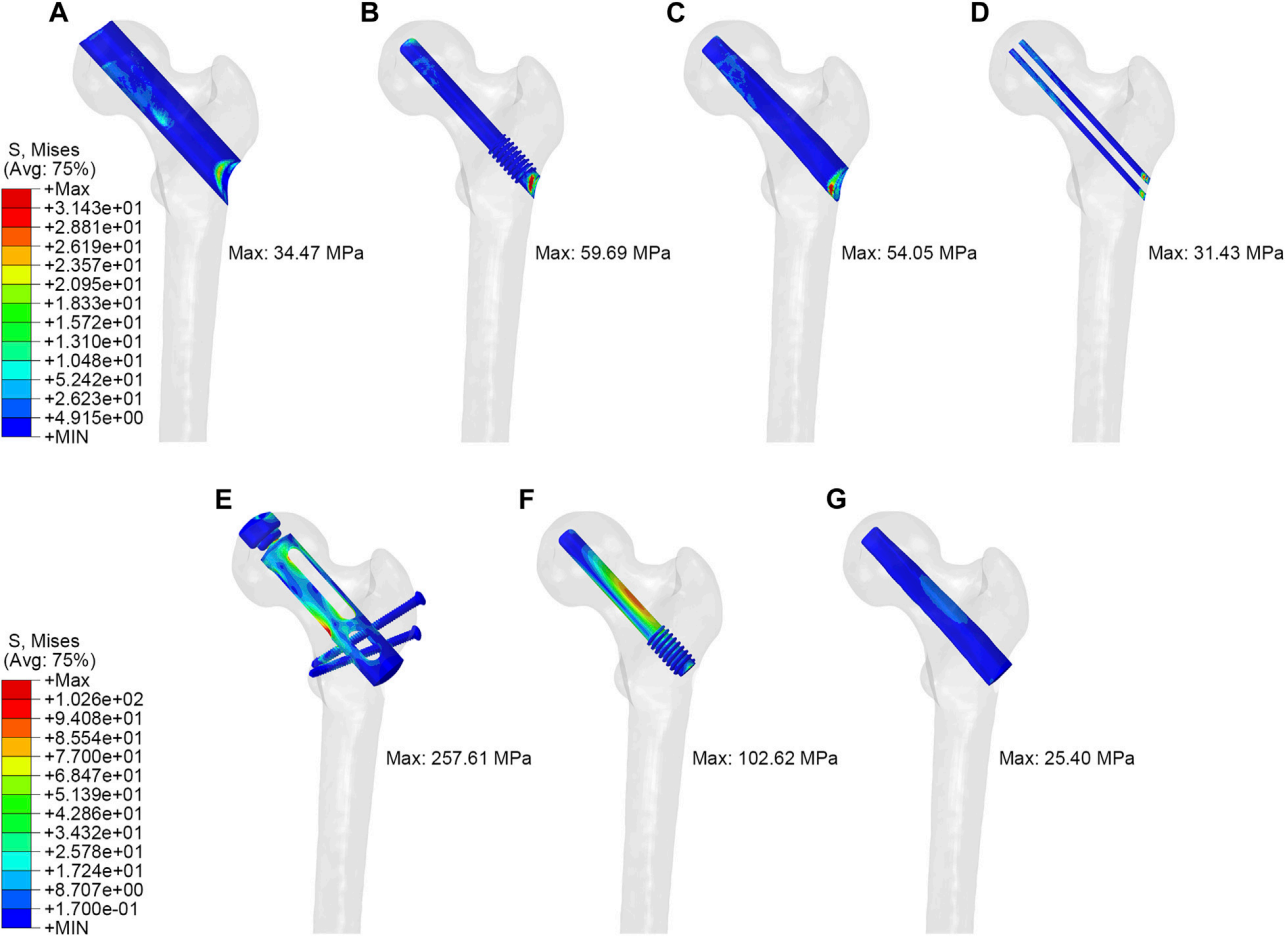
The von Mises stress distribution of tunnel and implant: **(A,E)** partial replacement prosthesis, **(B,F)** porous tantalum rod implantation, **(C,G)** fibula implantation and **(D)** simple core decompression.

As shown in Figure 3, in the simulation of normal walking gait, PRFH group had the lowest overall femoral stress value of 63.3 MPa, and the highest stress was found in the core decompression group, which was 96.8 MPa. For the most concerned stress of the femoral head region, the partial replacement group had the smallest stress of 22.8 MPa, followed by the fibular graft group 23.6 MPa, tantalum rod 33.4 MPa, core decompression 37.2 MPa. This study also measured the bone tunnel stress distribution, and the minimum stress occurred in the core decompression group, which was 31.4 MPa. The partial replacement bone tunnel stress was 34.5 MPa, only higher than the core decompression group, and decreased by 42.3% and 36.3% compared to tantalum rod and fibular groups, respectively. Figure 5 illustrated the stress distribution of the partial replacement prosthesis, tantalum rod, and fibula. In the PRFH group, the von Mises stress of the prosthesis was the highest, reaching 257.6 MPa, exceeding the CDFG group (102.6 MPa) and CDTRI group (25.4 MPa). The maximum stress of the partial replacement prosthesis occurred at the junction of the prosthesis head and neck. The peak stress of the tantalum rod and fibula groups occurred in the central region of the implants, measuring (102.6 MPa) and (25.4 MPa) respectively. The partial replacement prosthesis bore more stress transmitted along the proximal femur, yet this von Mises stress value was far below the yield strength of titanium alloy (850–900 MPa). The von Mises stress cloud of four groups were shown in Figure 4 and Figure 5.

### Displacement distribution

**FIGURE 6:**
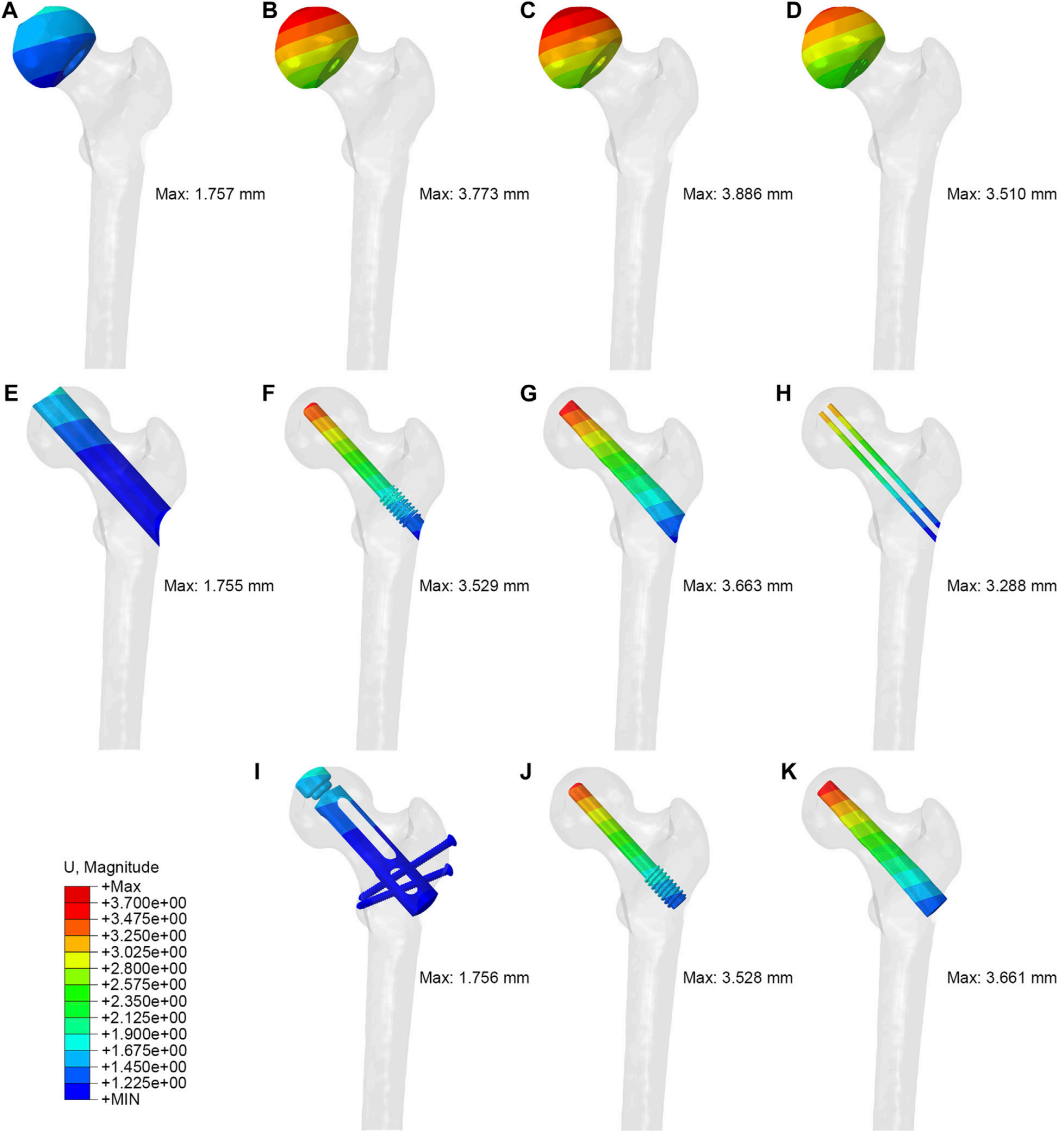
The displacement distribution of femoral head, tunnel and implant: **(A,E,I)** partial replacement prosthesis, **(B,F,J)** porous tantalum rod implantation, **(C,G,K)** fibula implantation and **(D,H)** simple core decompression.

The maximum displacements of PRFH, CDFG, CDTRI and CD groups were 1.757 mm, 3.773 mm, 3.886mm, 3.510 mm, respectively. This indicates that the partial replacement prosthesis group was the most stable. At the same time, in the three models with implants, the maximum displacement of the partial replacement prosthesis was 1.756 mm, which was 50.2% and 52.0% less than tantalum rod and fibular groups, respectively. The maximum displacement of PRFH group’s femoral head was reduced by 53.4, 54.8% and 49.9% compared to CDTRI, CDFG, CD groups, respectively. The displacement distribution of four groups were shown in Figure 6.

## Discussion

The optimal hip-preserving treatment remains a subject of controversy in patients diagnosed with osteonecrosis of the femoral head (ONFH) at ARCO stages II and III, particularly in stage III cases accompanied by femoral head collapse. This study presents, for the first time, the precise treatment of stage III ONFH using the “substitute the beam for a pillar” technique and performs a comparative finite element analysis with other hip-preserving procedures, specifically: partial replacement of the femoral head (PRFH), core decompression (CD), core decompression with fibular grafting (CDFG), and core decompression with tantalum rod implantation (CDTRI). The results showed that the PRFH prosthesis restored skeletal integrity and provided stable support, which reduced load and stress concentration on the femoral head, lowering stress levels and the risk of collapse.

The fundamental concept underlying hip-preserving surgery centers on decompression. CD entails the creation of perforations to alleviate pressure within the femoral head, yielding benefits such as enhanced blood circulation, mitigation of inflammatory responses, stimulation of new bone formation, and the achievement of pain relief. The integration of CD with tantalum rod implantation and fibular implantation (either vascularized or non-vascularized) provides structural reinforcement to the femoral head during decompression, thereby aiding load distribution and diminishing stress concentration. However, due to constraints inherent in surgical design, instrumentation, and technology, the initial surgical approach has been associated with relative operational complexity and suboptimal outcomes (Olsen et al., 2016; Zhang et al., 2022): 1) Complete necrotic bone removal is hindered by instrument limitations; 2) exclusive reliance on bone flap implantation proves inadequate in adequately supporting early postoperative mobility; 3) the procedure’s extensive and technically demanding nature contributes to pronounced donor-site morbidity and protracted rehabilitation periods; and 4) most crucially, for patients with femoral head collapse at ARCO Stage IIIB due to osteonecrosis, these techniques fail to attain satisfactory therapeutic efficacy.

This study reveals that, in term of displacement cloud map, the treatment of femoral head necrosis with PRFH enhances the overall structural stability of the femur by 49.9%–54.8% compared to the other three techniques. This is manifested by a commendable ability to withstand both compressive and tensile forces. Within the femoral model, the lowest von Mises stress extreme occurs in the PRFH group, measuring 63.3 MPa, which translates to a reduction of 16.9%–34.6% compared to alternative surgical approaches. Hence, the design of our PRFH technique, involving the removal of necrotic femoral head portions along with collapsed cartilage, facilitates the restoration of the femoral head’s surface structure. This optimization of overall mechanical performance significantly diminishes the overall stress distribution within the femur. In this study, the prosthesis partially shared the pressure on weight-bearing area, which significantly reduced the stress of the femoral head and proximal femur. The stress reduction was more pronounced than in the other three groups. The peak von Mises stress of the femoral head and proximal femur in the PRFH group were 22.8 MPa and 37.4 MPa, respectively, which were 3.1%–38.6% and 12.8%–37.4% lower than those of the other three surgical methods.

The other three methods (CD, CDFG, and CDTRI) had some limitations in biomechanical aspects. Simple core decompression (CD) has become the reference technique widely used in patients with early-stage ONFH Since popularized by Hungerford (Marker et al., 2008). But the results of CD are always debated and controversial (Koo et al., 1995; Zalavras and Lieberman, 2014b). Some studies (Hong et al., 2015; Liu et al., 2021) questioned and reported that, in fact, CD was not superior to non-surgical treatment, it cannot repair the femoral head which even lowers its biological strength and causes collapse. They found that when there is a subchondral fracture (47% in ARCO stage 3), compared with the pre-collapse stages (85% in ARCO stage 1% and 65% in ARCO stage 2), the success rate of core decompression is even worse (Mont et al., 2015). CD only relieved the intraosseous pressure in the necrotic area, but did not provide structural support or stimulate bone regeneration. CD did not prevent the progression of ONFH, and might even accelerate the collapse of the femoral head due to stress redistribution.

To address this problem, researchers applied vascularized and non-vascularized bone grafts or combined biomaterials such as tantalum metal before femoral head collapse. Nevertheless, the results of treatment outcomes reported from published studies were not consistent. The addition of non-vascularised bone-graft can provide structural support to the subchondral plate, however, procedures often require wide surgical dissection and hip dislocation with associated morbidity (Mont et al., 1998). While free vascularised fibular grafting addresses structural concerns associated with core decompression, it too requires an extensive and technically demanding surgical procedure and is associated with high donor-site morbidity and prolonged rehabilitation (Vail and Urbaniak, 1996; Louie et al., 1999). Reported failure rates following core decompression and tantalum rod implantation have ranged from 2% at 15 months (Liu et al., 2010) to 56% at 18 months (Floerkemeier et al., 2011). In addition, the risks of rod removal include tantalum debris, increased operation time, blood and bone loss, and increased risk of femoral fracture (Fernández-Fairen et al., 2012; Owens et al., 2012).

According to some studies, there is a high rate of retained tantalum debris on post-operative radiographs after total hip arthroplasty following failure of core decompression and tantalum rod implantation (Mont et al., 1998). This debris on the articular and the implant may have long-term effects (Olsen et al., 2016). According to the findings of histopathologic retrieval analysis conducted by Tanzer et al. (Tanzer et al., 2008), possible reasons for these results could be that the porous tantalum rod provided insufficient mechanical support for the subchondral bone of the necrotic area, and that there was no occurrence of bone regeneration in the necrotic area. Findings from multiple studies have reported on failure after porous tantalum rod insertion in a large number of patients, and conservative management is not an option for these patients. Thus, conversion to hip arthroplasty is the preferred treatment in most cases.

The limited lifespan of the replacement femoral head causes young patients to enter an endless cycle of repetitive revision once hip arthroplasty is chosen. Partial replacement prosthesis has a bionic porous structure that enhances the bond between the prosthesis and the surrounding tissue. The hollow structure bone graft within the partial replacement prosthesis promotes the biological fixation of the prosthesis and the surrounding healthy bone tissue, improving the stability of the prosthesis. The porous structure also alleviates the stress concentration at the interface of the prosthesis and the host bone, preventing prosthesis loosening or fracture. Moreover, this bionic structure partial replacement prosthesis can better adapt to the morphology and mechanics of the proximal femur, reducing the mismatch and stress difference between the prosthesis and the host bone, and improving their biocompatibility and mechanical stability. The peak value of the bone tunnel was measured to be 34.5 MPa, which was only lower than that of the core decompression group. Additionally, this reduced the risk of fractures around the prosthesis. The partial replacement prosthesis selectively replaces the necrotic segment of the femoral head, maintaining the integral bone and soft tissue structures of the proximal femur and hip socket. It is equipped with effective extraction instruments, ensuring minimal interference with total hip arthroplasty surgery. Therefore, partial femoral head replacement can serve as a transitional or bridging procedure for total hip arthroplasty, offering more options for young or active patients.

### Research limitations and future research directions

Our study has some limitations that need to be acknowledged. First, in our study, the “ideal position” is explicitly defined as the weight-bearing region of the femoral head. If the site of femoral head necrosis is not in the ideal position and the position of the partial replacement prosthesis changes, it may potentially affect the finite element results. Second, we used a linear elastic material model for bone tissue, which might not capture the nonlinear behavior of bone under large deformation or damage. Third, we used a static loading condition for gait cycle, which might not reflect the dynamic loading condition *in vivo*. In this study, point loading force was applied, which may influence the calculation results, especially when the loading point is close to the ONFH site. On the other hand, the femur model we constructed did not take into account articular cartilage, which could affect the results. Additionally, long-term follow-up clinical trials are necessary to assess the stability, hip function and lifespan of partial femoral head replacement.

## Conclusion

In conclusion, our study compared four surgical methods for ONFH using finite element analysis: PRFH, CD, CDFG, and CDTRI. The results showed that PRFH had the best biomechanical performance among the four methods, as it reduced the stress concentration and displacement in the necrotic area, increased the stress transfer to the healthy bone tissue, maintained the shape and stability of the femoral head, and restored the normal biomechanics of the hip joint. Our findings suggest that PRFH is a superior surgical method for ONFH in terms of biomechanical effects.

## Data Availability

The original contributions presented in the study are included in the article/Supplementary Material, further inquiries can be directed to the corresponding authors.
